# When filth became dangerous: the miasmatic and contagionistic origins of nineteenth-century cleanliness practices among Swedish provincial doctors

**DOI:** 10.1017/mdh.2024.34

**Published:** 2025-01

**Authors:** Annelie Drakman

**Affiliations:** Stockholm University, Stockholm, Sweden

**Keywords:** Sweden, Nineteenth-century medicine, Hygiene, Dirt, Miasma, Contagion

## Abstract

This investigation sheds light on the social history of pathogenic dirt and its significance for shaping medical practices during the nineteenth century. It consists of an analysis focusing on Swedish medicine, using 8800 yearly reports written 1820–1900 by Swedish provincial doctors for the National Board of Health in Stockholm. The main argument is that the provincial doctors’ perceptions of the relationship between dirt and health during this century can be better understood by focusing on similarities in the handling of different kinds of pathological dirt over the course of many decades, rather than seeing interest in cleanliness as something mostly unprecedented. A novel cleanliness regime became dominant during the latter third of the century, meant to counter a new hybrid between everyday dirt – bodily emanations from healthy bodies – and matter believed to have caused miasmatic and contagionistic disease. New ideas about filth and its impact on health played a crucial role in the development of public health and sanitation movements, and were a precondition for everyday dirt becoming a central medical problem around the turn of the twentieth century, but as is shown, they built on old precedents. Thus, the miasmatic and contagionistic approach to disease shaped conceptions of hygiene and cleanliness.

How did filth become the object of so much medical attention during the nineteenth century? Changing conceptions of what constituted dirtiness, and how uncleanliness affected health – the social history of pathogenic dirt – were at the centre of some of the most profound transformations of medical theory and practice during this century, such as the expansion of public health, and the establishment of the sanitary and hygienic movements.^1^ Filth being reformulated as a medical problem during the nineteenth century caused a great expansion of the medical professions’ responsibilities, areas of interest, and reformational activity, and motivated the establishment of new organisations, changes which aided processes of professionalisation.^2^

In this study, I analyse reports written by hundreds of Swedish provincial doctors between 1820 and 1900 for *Sundhetscollegium* [*The National Board of Health*] in Stockholm. I have mostly relied on the corpus of 8800 transcribed versions in the online database *Medicinhistorisk databas* [*Database of Medical History*], but have also made use of some forty originals at Riksarkivet [The National Archives] in Stockholm.^3^

The goal of this article is to show how the cleaning practices of this group of doctors shifted during the nineteenth century; what kind of substance they considered to be dirt, how it was removed, and how it was understood to affect health. By ‘dirt’, I mean all physical matter provincial doctors set out to destroy, remove, or render harmless through cleaning practices. Cleaning is here defined as the processes through which undesirable matter is removed in the following ways: washing (letting water flow over a body or an object to flush away matter), airing (letting fresh air into a room to disperse matter), rubbing (using friction to remove matter), fumigating (burning odorous substances to destroy or overpower airborne matter), or any other method which the doctors themselves called ‘cleaning’.^4^ In the following, I will argue that the process where dirt began to be seen as a medically harmful substance partly resulted from an amalgamation between everyday dirt – filth amassed through regular living by healthy people – and the processes of handling miasmatic and contagionistic matter in the early nineteenth century. As this study will show, countering epidemic disease spread through miasma or contagion – the two dominant theories of disease dissemination before the 1870s in Western medicine – in practice often meant cleaning away specific matter.

In several ways, the case of Swedish provincial doctors affirms established historiographies. As historian Christopher Hamlin and others have indicated, conceptions of cleanliness and their impact on health profoundly influenced how propriety, disease, and the appropriate relationship between state and subject were understood in many countries around the turn of the twentieth century.^5^ The Swedish case shows this well-studied process from a new angle. Sweden was a mostly rural country at the periphery of trans-Atlantic medical discourse, and this case gives a fresh perspective to a topic mostly studied regarding urban transformations in the United Kingdom, France, and the United States. Swedish provincial doctors as a group also merit research. Their centralised education, status as employees of the state, links with international networks of publishing and correspondence, extensive commentary on the homes, clothing, and bodies of their patients, and the regular nature of their reports, make these doctors a fascinating lens through which to study wider phenomena such as the increased interest in the environments of the poor, and the influence of bacteriology and the sanitary and hygienic movements on medical practice.

There has been much excellent historical work on the growing interest in cleanliness in Europe and the United States during this period. Generally, the story is told this way: Dirt was of little concern to the medical community, national governments, and the general population alike in the eighteenth century.^6^ This changed during the early nineteenth century when waves of epidemic disease (primarily cholera) gave new urgency to the old theory of miasma: the idea that decomposing organic matter spread disease though noxious vapours.^7^ The impetus to remove the material origins of miasmatic contagion helped establish the sanitary movement, which mainly concerned itself with remedying urban filth, by for instance regulating waste removal and building sewers.^8^ Other changes also led to an increased focus on dirt and its impact on health during the nineteenth century, such as the growing awareness of the adverse influence of industrial emissions, and the focus on animal pests and toxins. As David Arnold has shown, conceptions of poison shifted, and the seriousness of the threat from toxic influences was increasingly discussed as a dangerous problem during this period, both in India and Europe, leading to a level of interest which, in Arnold’s memorable phrase, ‘at times bordered on an apocalyptic “fear of a poisoned world”’.^9^

The same increased attention was true for many other concepts of disease and symptom clusters (such as ‘summer diarrhoea’) due to changes related to bacteriological advances from the 1870s onwards, and developments within organic and inorganic chemistry.^10^ Great work has been done on how these changes happened, showing that they were seldom caused by open conflicts, but rather were adaptive responses to practical problems. This perspective on change as additive and gradual is also demonstrated by Michael Worboys, who shows the proliferation of different germ theories in Britain during the last third of the century as at least partly driven by practical problem solving, for instance by surgeons trying to avoid sepsis, and veterinarians struggling to treat cattle plague.^11^

These changes in what matter was seen as dangerous to health helped transform the sanitary movement into the hygienic one, more concerned with everyday cleanliness on a household level, although David Barnes convincingly argues for a sanitarian-bacteriological synthesis rather than two separate movements.^12^ These movements also helped change conceptions of dirt, for instance by discussing ‘filth-diseases’ (the sanitarian reformer John Simon’s term), such as typhoid.^13^ Christopher Hamlin reminds us that before sanitary science, many people’s drinking water was hardly better than ‘diluted sewage’.^14^ This increased focus on cleanliness affected a wide array of practices, for instance encouraging a stronger state oversight of food production and handling in Sweden.^15^ That contaminated food was increasingly seen as the origin of epidemic outbreaks, mainly of tuberculosis, certainly also affected conceptions of dirt as pathogenic.

Thus, as historians have shown, the belief that dirt threatened health injected urgency into public health initiatives and social reforms. This had profound effects on medicine as a discipline. Both hygiene as a science, and medical bacteriology in their beginnings focused on dirt, mainly to understand sepsis and putrefaction.^16^

The growing faith in the health benefits of cleanliness also brought medicine and public health closer together especially as the sanitary and hygienic movements often operationalised removing dirt as the practical means of combatting more elusive entities such as germs and bacteria.^17^ As Nancy Tomes and others have shown, profound social changes were enacted around the turn of the twentieth century by means of household hygiene, which was transformed by new conceptions of cleanliness promoted in a multitude of venues such as cooking manuals, syllabi, and advertisements.^18^ Conceptions of dirt and cleanliness also lay at the centre of histories of personal hygiene and notions of healthy skin.^19^ In conclusion, many important societal changes can be considered to stem from a reformulation of the pathogenic properties of dirt.

With this article, I aim to reframe one specific aspect of the historiography of pathogenic dirt. Rather than assuming that a new connection between dirt and disease arose at the end of the nineteenth century, I will show that this link had already existed for many decades through the practical handling of matter believed to cause and contain miasmatic and contagionistic disease, and rather was *re-conceptualised* into also defining how everyday dirt should be considered and handled.

## Swedish provincial doctors and their annual reports

The nineteenth century was a period of dramatic social and political transformations, but during this time, Sweden was characterised by ethnic and religious homogeneity, without major disruptions from natural disasters, political revolutions, or, after 1814, war. It was a sparsely populated country, run by a paternalistic state with firm control over public health and medicine. The medical system operated through a centralised bureaucracy with doctors acting as civil servants.^20^ In some respects, the Swedish example mirrors those of nineteenth-century Europe and America, for instance in the impetus that cholera pandemics gave to the establishment of sanitary movements. However, during the nineteenth century (in contrast to the twentieth, when the Swedish hygienic movement had strong ties to the welfare state), cleanliness reform was largely advanced by local initiatives from state officials. Here, provincial doctors were a crucial category of actors.

The post of provincial doctor was established by royal command in 1688 and existed until 1973.^21^ They were state-appointed general practitioners and local administrators, stationed in districts covering the country. Most districts contained tens of thousands of inhabitants, i.e. potential patients, as there was little competition in the medical marketplace outside of the larger cities in Sweden during the period (a marked difference from several other countries). Swedish provincial doctors are often described as rural physicians, but the distinction between rural and urban practice is vague.^22^ Although the provinces were predominantly rural, there were provincial doctors stationed in all major cities, including Stockholm, throughout the century.^23^ However, Sweden was relatively sparsely populated, and lacked large urban areas – Stockholm, the most populated city, had only 100,000 inhabitants in 1856. Until at least the 1860s, many provincial doctors lived in the largest local town, which often gave the district its name. Consistently, and unlike their counterparts in other countries, provincial doctors shared similar concerns and attitudes towards dirt across urban and rural contexts.^24^

Each provincial doctor had three chief responsibilities: to prevent and treat illness among the general population, to oversee other medical practitioners locally, and to gather information for *The Department of Health* in Stockholm.^25^ They ran their own medical practices, charging a fixed patient fee set by the state. They also inspected midwives, oversaw vaccinations, were expected to prevent and handle epidemics and outbreaks of venereal diseases, assisted veterinarians, performed forensic examinations, inspected mineral wells, pharmacies, and young men for the draft, among many other tasks.

In the course of their duties, the doctors both accepted patients into their own homes and visited them, most commonly in association with outbreaks of epidemic disease. Patients included members of the local gentry and bourgeoisie, as well as a small but growing group of industrial workers. Primarily, however, they were peasants. The social background of provincial doctors themselves varied, but according to professional biographies listing their fathers’ professions (e.g., captain, prosecutor, consul), they were mainly from the bourgeois middle class.^26^ A marked class difference thus existed between them and the majority of their patients.

Provincial doctors had intimate contact with patients’ bodies, clothing, and homes, giving them plenty of opportunities to observe cleanliness practices (or the lack thereof) up close. The range of their responsibilities, from patient care to public health, gave them a particular perspective on the issue of dirt and cleanliness, although this did not diverge from that of their patients until the second half of the century when complaints about the filthiness of peasants became common in their reports. This was related to expanding responsibilities as state representatives and changing conceptions of cleanliness among doctors. Religion does not seem to have been a factor in this shift. The state church of Sweden was Evangelical Lutheran; however, provincial doctors rarely mentioned religion.^27^

Most Swedish doctors from the same generation would have known each other since they had been educated together at the universities in Uppsala or Lund.^28^ Unlike other countries, during the nineteenth century, Swedish medical education was firmly regulated by the state.^29^ Changes in the curriculum, incorporating for instance bacteriological advances from the 1880s onwards, affected doctors’ responses to dirt, as did hygiene instruction, which was mandated from the 1890s.^30^ All Swedish medical students spent at least a few months at the *Karolinska Institutet* in Stockholm, which provided the only surgical education available in the country. The English surgeon Joseph Lister’s new antiseptic methods were very influential there from the 1870s onwards.^31^ Thus, the doctors’ shared outlook was shaped by their centralised education. It was further supported by networks of letter writing, official reporting, and journal subscriptions, both nationally and internationally.

From the 1860s, annual meetings of provincial doctors were held around the country, fuelled by a growing sense of professional identity, and in 1881, the *Swedish Association for Provincial Doctors* was formed.^32^ This association was in frequent contact with individual provincial doctors, including those who were geographically isolated, in a lively network of knowledge exchange. Their example can therefore cast light on a broader medical discourse – they were not isolated.

Beginning in 1755, provincial doctors were required to submit annual reports describing the past year’s activities and the state of health in their district to the *Collegium Medicum*, later *The National Board of Health.* ^33^ These reports were an important information-gathering tool of the Swedish state, which had pioneered the assembly of demographic statistics since the mid-eighteenth century.^34^ We might expect these reports to have an administrative focus, but instead many go into extensive detail on the doctors’ own actions, cases, and medical beliefs. Provincial doctors designed their own reports until 1851, when a template was introduced, requesting information about the weather, common diseases, and the doctors’ relationship to local institutions such as parish councils, health police, poor relief and prisons, vaccinators, public schools, pharmacies, midwives, spas, baths, and military personnel.^35^

The official template notwithstanding, the perspectives expressed in these reports from doctors stationed across the country, in different rural/urban settings or climatological circumstances, are remarkably similar at any given moment. Some generational differences can be found (for instance, older doctors using the word ‘miasma’ more often than younger ones in the 1880s), but no regional difference of note. In this regard, my study differs from others that have focused on similar sources in other countries, emphasising regional differences. Evelyn Ackerman, investigating French rural medicine 1800–1914, limits her study to a single district, assuming that a wide variety of local traditions would make a national overview impossible.^36^ Pamela Gilbert, studying nineteenth-century American popular disease perceptions, states that they were locally rooted and regionally distinct, with large variations between different locations, levels of urbanisation, and ethnic groups.^37^ Such differences are difficult to find in reports from Swedish provincial doctors, despite the country being one of the largest in Europe, covering several climate zones.^38^ Rather, the major variations are chronological.

## The emergence of pathogenic everyday dirt

To clarify how the conceptions of filthiness of Swedish provincial doctors changed over the course of the nineteenth century, I will analytically separate what I call ‘everyday dirt’ from other kinds of matter considered to be filthy, although the doctors themselves rarely drew such a distinction. This category overlaps with twenty-first-century conceptions of dirt. It consisted of matter which accumulated on bodies and in homes from regular processes of living by healthy people. Materially, it consisted of discarded organic and inorganic matter, like food scraps, and bodily waste from healthy animals and humans. It also included secretions accumulated on an unwashed body, such as sweat and emanations like exhaled air.^39^ This dirt could be seen (discoloured clothing), smelt (disgusting stench), and felt (sticky floors). If it was not perceived by the senses, it was not considered to be present. To clean everyday dirt meant removing it by washing it with water, sweeping, and airing out rooms until its presence could no longer be sensed. Provincial doctors considered this substance mostly unimportant during the early nineteenth century, and it was seldom discussed as medically relevant. Filthiness was not described as a medical problem, and doctors only rarely described engaging in or ordering cleaning to counter everyday dirt. This lack of interest is observable both quantitatively and qualitatively and corresponds to a lack of concern that has been well charted by historians working in other countries, such as France.^40^

For instance, it is easy to track when specific words were first used in *Medicinhistorisk databas*’s collection of 8800 reports from 1820 onwards.^41^ The most common Swedish words related to cleanliness and filth were not used here until the 1830s. The Swedish word for ‘dirt’, ‘smuts’, does not appear until 1835. The word ‘osnygg’, meaning ‘unkept’, was used for the first time in 1843. The word ‘snygg’, meaning ‘orderly, well-kept’, occurred for the first time in 1851. The word ‘snusk’, meaning ‘filth’, can only be found from the 1850s onwards, and ‘prydlig’, meaning ‘neat’, occurs for the first time in 1852. The word ‘renlig’, meaning ‘inclined to clean’, was used only twice before 1850, but forty-eight times between 1850 and 1900. The word ‘tvätta’, meaning ‘to wash’, is used sixty-five times in the database, only once before the 1840s. The word ‘dirt’ was used twice in the 1830s and forty times in the 1890s.^42^

That provincial doctors rarely used words describing cleaning during the early nineteenth century should not be seen as conclusive proof of disinterest. However, other indicators point the same way, such as a shift in the meaning of words. Before the 1860s, two words which in contemporary Swedish are firmly associated with cleanliness, ‘smuts’ (‘dirt’) and ‘oren’ (‘unclean’), were not used to describe cleaning in the reports. Before 1860, the term ‘oren’ only describes tongues with an unusual colour, a symptom of disease.^43^ After this date, the word instead indicated a perceived lack of care for bodies and homes. Before the 1850s, the word ‘smuts’ only described weather conditions, like rain making a road muddy, or else the colour ‘dirt yellow’.^44^ In the late 1800s, however, it was consistently used to describe insufficient cleaning of dwellings and bodies.

In the rare cases when cleanliness was mentioned in the early years, doctors mainly used it to discuss social conformity. Cleanliness indicated good character. In 1851, the doctor in Jönköping described the population of his district as ‘reliable, industrious, clean, honest’.^45^ Labelling patients as clean could also prove that a disease was not self-inflicted.^46^ Uncleanliness, conversely, was associated with idleness, as when the doctor in Norrtälje in 1859 stated that ‘Inland peasants … are lethargic and very unclean’.^47^

The presence of everyday dirt could also be described as rudeness or lack of respectability. For instance, in the 1850s, Dr Dahlberg in Söderåkra wrote that he could excuse male farmers for neglecting the neatness of their hair and beards, leaving their hands soiled and their faces dirty while working, but he could not forgive their wives at home, who, ‘with the exception of the beard, look just the same’.^48^ The presence of everyday dirt was thus used to indicate divergence from conventions of orderliness. ‘Dirty’ as an epithet was primarily used to illustrate a person’s lack of social adaptation, working capacity, and obedience. During this period doctors described everyday dirt as a social affront or a nuisance, not as a health hazard.

Everyday dirt could even, in rare instances, be presented as medically beneficial. During the first half of the century, both provincial doctors and their patients generally considered the body to be permeable, with weak defences against harmful environmental influences.^49^ In that context, dirt could be understood as an encapsulating shell keeping the vulnerable body warm and safe.^50^ In 1850, the doctor in Wrigstad wrote:

Notwithstanding my instructions about the importance of cleanliness, young men who are to be inspected for the draft appear as filthy, as if they have been pulled out of a swamp. Therefore, I must sometimes use an instrument to scrape off the dirt from their extremities in order to inspect them. When I ask why they do not bathe, they say ‘to protect themselves from the cold’.^51^

Thus, removing everyday dirt could be perceived as dangerous, since it meant getting rid of a coating substance which protected the body.^52^ In the rare cases when provincial doctors did state that everyday dirt harmed health during this period, they gave a specific reason: it clogged the skin’s pores. In 1858, the doctor in Vimmerby described everyday dirt as a medical problem in this way: many peasants had ‘a large part of their bodies covered by a bark of dirt. This bark interferes with the crucial function of the organism … by which I mean the discharge of substances through the tallow and sweat glands. Thus, harmful substances are retained in the blood, causing disease’.^53^

Alain Corbin describes similar views among French doctors in the early nineteenth century: ‘Dirt obstructed the pores; it held back the excremental humours, favoured the fermentation and putrefaction of substances; worse, it facilitated the “pumping back of the rubbish” that loaded the skin’.^54^ Everyday dirt potentially prevented the ejection of harmful substances from the blood. But overall, during the early 1800s, it was only considered a medical problem in exceptional cases.^55^

## Medical problems in the early nineteenth century: handling contagious and miasmatic matter

When provincial doctors described their own cleanliness practices during the early nineteenth century, they generally connected them to their attempts at preventing the spread of epidemic disease. Their practices were generally based on the two predominant explanations for disease transmission during the period: the contagionistic and miasmatic theories. In practice, this meant working to manage harmful material substances: the material manifestations of contagion and miasma.

The contagionistic and miasmatic (or anti-contagionistic) theories of disease transmission have been studied as connected to political considerations.^56^ Historians have also explored the processes by which the object of cleanliness practices changed from decaying matter to mundane dirt in the nineteenth century. However, such studies generally explain these changes through circumstances exterior to medical practice, for instance by showing how the religious beliefs of British sanitary reformers framed disease as divine punishment for sinful squalor.^57^ However, contagion and miasma could indeed serve as abstract principles explaining how disease was transferred, but medical practitioners also used them as a practical means of identifying the presence or absence of specific pathogenic matter, and thus to decide when and where to clean. Doctors managed miasma and contagion as though they were tangible substances present in a particular place, and could be removed or rendered harmless through cleaning practices such as washing, airing, sweeping, or fumigating. As medical problems, they were often solved by cleaning.

## Cleaning to counteract contagion

During the early nineteenth century, while provincial doctors largely disregarded everyday dirt – body fluids and emanations from healthy persons – they often focused on removing physical matter which had been generated by those who were ill. Such substances (for instance spit, faeces, and exhaled air) were considered to be the concrete matter which mediated infection. Doctors assumed that contagious matter was present in places where sick people had stayed, which prompted the cleaning of sickrooms during or after an illness. According to contagionism, infectious diseases are transmitted by an undefined disease agent moving from person to person.^58^ In practice, this agent was considered to be present in bodily waste and emanations.

Thus, in the first half of the century, Swedish provincial doctors primarily prescribed or engaged in cleaning during the illness, recovery, or after the death of a patient. When cleaning practices were discussed, they were almost exclusively prescribed as a reaction to a specific event rather than a regular, daily practice. Sick rooms were cleaned after, not prior to, patients’ arrival. For example, while treating a person suffering from fever in 1843, the doctor in Vimmerby ordered a ‘thorough cleaning of the bed and the sickroom’.^59^ Thus, the room was considered to have become ‘medically’ contaminated through the secretions of the patient.

Most aspects of this reactive category of cleanliness remained constant between 1820 and 1900: the substance which needed to be removed (the contagious bodily excretion), the moment when cleaning was performed (during or after the occurrence of disease, rather than before it), and the place (the room where a sick person had stayed). However, two new methods of destroying and identifying contagious matter were introduced towards the end of the century: disinfection and bacteriological tests.^60^ Of course, these new methods are connected to well-known discoveries and inventions within the history of medicine, such as the breakthrough of bacteriology from the 1870s onwards. Many Swedish doctors were well aware of cutting-edge medical research, through their education and by subscribing to international medical journals such as *The Lancet.* ^61^ After the 1840s, most of them also subscribed to the Swedish medical journal *Hygiea*, which doubled as a sort of bulletin board for this group, and after 1877, *Eira, Journal for Practical Medicine.* ^62^ Both carried translations from the most important French, German, and British medical journals.^63^ The reception of international breakthroughs varied, but the overarching pattern was the rapid adoption of new techniques and therapies. For example, the first Swedish vaccination was carried out less than two years after Edward Jenner’s article describing the practice was published in June 1798.^64^

What matters here is that before and after the breakthrough of bacteriology, doctors’ practices stayed mostly unchanged, structured by the same basic idea: the bodily waste emanating from a sick person could spread disease, and should thus be removed by cleaning.

One crucial feature of contagious matter, which distinguished it from everyday dirt and miasmatic matter, was that the contagious agent could be imperceptible yet still present. To the provincial doctors, seemingly clean surfaces could be hiding indiscernible contagion. Thus, they could not rely on their eyes or noses to determine whether a place contained contagious matter. In 1897 the provincial doctor in Dorotea warned ‘there is … plenty of water which is fresh and tastes good to drink, but as the wells are placed close to outhouses and barns, it ought to be far more contaminated than what its taste or smell suggests’.^65^ From the 1890s, laboratory tests became a popular method for disease identification among Swedish provincial doctors, who enthusiastically adopted bacteriology as a conceptual framework.^66^ However, throughout the nineteenth century, the main method of identifying the presence of contagious matter was that people had fallen ill after being in contact with it. For instance, in 1900 the doctor in Nyfors wrote:

Last year, I was called to a home where after 14 days of diphtheria, one child had died…. The rooms were disinfected, the family moved away, and another family moved in. Everything was quiet until December of this year. As the mother wanted to clean her house in preparation for Christmas, she poked into and disturbed every last corner of the room. This probably awoke old, dormant germs, because the next day, the mother and all her six children fell sick with diphtheria. Obviously, the disinfection had not been extensive enough.^67^

Thus, contagion could lie dormant, invisibly, for years. Contagion was not connected to messiness, stickiness, or untidiness in a particular room, nor was it a sign of disorder or laziness; in the quote above, it was reactivated through the virtuous activity of cleaning. The occurrence of disease was taken to prove that contagious matter was indeed present, even if it had not been identified. It was known only through its effects.

In summary, the handling of bodily excretions considered to be contagious was governed by three principles. First, contagion occurs after a particular event: the presence of a person with a contagious disease. Thus, it was eliminated through cleaning during or after a period of illness, never before, and it did not require recurring, daily cleaning. Second, contagion could be present even though it could not be perceived by the senses. Third, it was very dangerous, potentially causing illness or death. During the late 1800s, all these qualities would combine with a category of dirt from which, earlier in the century, it had been completely distinct: everyday dirt.

## Methods for countering contagion begin to be used for handling everyday dirt

During the early 1800s, the kind of matter which was created through illness and mediated contagion, and everyday dirt which arose from healthy living processes, were considered to consist of similar matter: bodily waste and emanations. But they were also seen as utterly dissimilar, having different effects and occurring in different circumstances. The perceived urgency in countering them was also distinctive. Removing the first was a matter of life and death; removing the other was an aesthetic choice, mainly just the polite thing to do.

This all changed during the last third of the nineteenth century when I argue that everyday dirt came to be seen as the direct cause of contamination. People covered in everyday dirt began to be perceived as potential sources of disease precisely because they carried the matter which now was considered to be the material manifestation of infection, *whether they had been ill or not.* The personal uncleanliness of healthy people became seen as directly hazardous to other people’s health. If dirty people could be persuaded to clean themselves, this danger would be radically reduced. Thus, methods for countering contagion and methods for cleaning everyday dirt were combined in a novel way, beginning around the 1860s, a change that concerned domestic spaces as well as bodies. In 1881, the doctor in Naum addressed this new hybrid while explaining that the messiness in healthy peasants’ homes bred contagion:

[disease is caused by] the peasants’ habit of storing food of all kinds, both solid and liquid, in the bedroom, and by the fact that one finds left-over food on the upper shelves of cupboards while sweaty socks and unclean clothes are stored on the lower shelves. This means their bedrooms are hotbeds for all kinds of contagious diseases.^68^

The problem was that clothes soiled by bodily emanations from healthy individuals were not kept separate from food, which could thus become contaminated. Everyday dirt – like sweat on socks – was now assumed to generate and strengthen infectious disease.

## Countering miasmatic disease through cleanliness

During the early 1800s, Swedish doctors, like their colleagues in other countries, were concerned with countering the spread of miasmatic disease. Miasma consisted of harmful, noxious vapours which emerged from specific material circumstances: the decomposition of rotting, fetid, or putrid animal or vegetable matter poisoned the air. Until the 1870s, miasma was one of the main causal explanations for the occurrence of epidemic disease.^69^ In the words of one provincial doctor, miasma was ‘matter undergoing changes detrimental to the normal continuation of animal and human life’.^70^ Doctors tried to counteract miasmatic, airborne influence by overpowering or destroying its foul smell, or by removing the matter which created it.

By historians, miasma is generally seen as an abstract theory of disease transmission, not as physical matter.^71^ However, the provincial doctors treated it as a polluting and contaminating material substance which could be remedied by cleaning.^72^ In the early nineteenth century, such substances were said to be created primarily in swamps and marshes when submerged vegetable matter rotted and its stench spread the process of decomposition to people who came in contact with it. Such bad air was often called ‘miasmatic vapours’ to emphasise that the air had been soiled by putrid particles. Some epidemic diseases, particularly malaria, were always assumed to be caused by miasma, but the concept was primarily used to explain the emergence of local epidemics when no chain of disease transmission from person to person could be found. The assumed presence of matter which might create miasma resulted in several different kinds of cleaning.

First, provincial doctors often took part in local initiatives aimed at reducing the amount of putridity in the environment by draining marshes and building ditches. This is similar to work done in other European countries as a part of the sanitary movement. For instance, the doctor in Katrinehamn in 1859 participated in a ‘systematic building of ditches throughout the entire municipality’.^73^ The point was to prevent and counteract stagnant water since a lack of circulation was seen as a necessary pre-condition for the processes of putrefaction.

Second, open wounds were rarely said to have been cleaned during the first half of the century, and if so, only when some aspect of putridity was apparent. Only one of the 40 provincial doctors’ reports in which the word ‘wound’ (‘sår’ in Swedish) was used between 1820 and 1859 describes the cleaning of wounds. The provincial doctor in Eskilstuna in 1820 used the word ‘wound’ twenty times in a single report, but wrote nothing about whether the wounds were cleaned.^74^ In the one instance where the cleaning of wounds was discussed in this period, by the doctor in Vimmerby in 1840, the doctor wrote that he had cleaned a wound only because the stench from it was ‘excruciating’.^75^ Thus, everyday dirt was not considered medically relevant regarding wound care during the period, but when putridity, as identified through smell, was present, cleaning was called for.

To prevent miasmatic disease the doctors also tried to destroy or overpower putrid smells, primarily by using smoke. During the first half of the century, having a room fumigated was a common part of the doctor’s visit. One indication that fumigation could be considered as a form of cleaning can be found in the word choices in a report from Eksjö in 1852, where the doctor wrote: ‘Whenever I arrive at a patient’s bedside, I order the rooms to be infused with smoke daily, and also that *cleanliness should be further pursued* by all other means available’.^76^

Miasma was believed to be airborne, and using smoke in a sickroom to clean the air made sense since smoke could change the properties of matter (for instance to preserve meat). Additionally, efforts were made to hide or destroy the smell of decay through stronger scents, like burnt coffee or vapourised vinegar. In this way, the doctors tried to thwart the penetration of miasmatic air into people’s bodies.^77^

That doctors cleaned in order to remedy miasmatic and contagious matter rather than everyday dirt until about the 1860s is also shown through another example. When the cholera hospital in Uppsala was equipped for 50 patients in 1857, its director purchased 10.5 litres of tar for the fumigation of hospital rooms. At the same time, he only purchased 425 grams of soap, less than 10 grams per patient.^78^ The relative quantities indicate that cleaning practices at the hospital were directed towards miasmatic and contagious influences, not towards everyday dirt, which was primarily removed through soap and water.

## The amalgamation of miasma and everyday dirt

Until the mid-1860s, miasmatic matter and everyday dirt were generally described as two separate categories, as in the following quote from the provincial doctor in Stockholm city in 1862: ‘The air is sullied chiefly in two different ways: 1. By exhalations from fermenting, decaying heaps of matter…. 2. through a poison of purely animal origin, generated when men live too closely together’.^79^ These ‘exhalations’ and the ‘poison’ were two distinct entities, formed in different ways. But towards the end of the century, the differences between them became blurred.

At first, the presence of everyday dirt was said to reduce bodies’ abilities to resist the damaging influences from miasma. Later, a hybrid category between the two was formed. From the mid-nineteenth century onward, airborne miasmatic influences were said to need ‘favourable conditions’ to be able to ‘take hold’ in a specific location.^80^ Everyday dirt was described as facilitating the effects of miasma, as when the doctor in Enköping 1853 stated that a cholera epidemic emerged when the categories of miasma and everyday dirt were combined: ‘[In cities] it ought to be easier for miasma to condense, while it will be more dispersed in the country-side, unless a great deal of humidity and animal filth is present, which I assume will work as a lightning rod for choleric contagion’.^81^ Thus, everyday dirt began to be conceptualised as kindling for disease. From the 1850s onward, in several different ways, doctors began to see a causal relationship between everyday dirt and disease outbreaks:

The miasmatic agent moves in different directions over the disease-infested town. It seems to become thinner and less harmful in places, where it is not fed by proper preconditions, such as the presence of uncleanliness, especially the kind of uncleanliness which consists of animal excrement…. Such [miasmatic] changes happen in the air when too many people live too close to each other in overcrowded, unkept living quarters, which are never cleaned.^82^

Everyday dirt – in the quote above described as animal excrement and changes in the air due to overcrowding – was now presented as something that attracted, bred, and encouraged disease. Everyday dirt had become directly or indirectly pathogenic.

Towards the middle of the nineteenth century, the term ‘miasma’ became more common in the provincial doctors’ reports, and was used to describe broader categories of decaying matter. Between 1820 and 1840 miasma was mentioned only in connection with swamps, and only rotting vegetable matter was said to generate it. But from the mid-1800s the descriptions changed, as human waste and household rubbish began to be depicted as potentially miasma-generating.^83^ When Dr Malmberg inspected the workers’ homes around the Motala factories in 1878, he declared that uncleanliness threatened their health:

Here, we find an excess of disorderly and disgusting outhouses, pigsties, piles of garbage and trash. The sight of this mess disturbs an orderly mind, and furthermore, this disdain for cleanliness is harmful in regards to hygiene. The foul vapours which emanate from here enter living quarters and permeate the local environment. During epidemics, such putrid emanations will severely harm the health of the population. Due to the high humidity locally, the emanations will be certain to create and sustain contagious matter.^84^

In this quote, everyday dirt harmed health. By putrefying, it became miasmatic and spread disease through foul vapours. Thus, miasmatic matter was no longer only caused by rotting vegetable matter in swamps, but could also emerge from human and animal waste – everyday dirt. In the same way, the provincial doctor in Uppsala in 1857 explained a cholera epidemic by stating that choleric miasma had been created through ‘the work of heat on animal excretions’.^85^ This new focus on excretions meant that the provincial doctors’ preventive work began to be directed towards urban environments and peasants’ homes rather than towards features of the landscape: ‘the most dangerous repositories for miasma can be found in courtyards and doorways’.^86^

This reconceptualisation of miasma from a natural process to a consequence of urban disorder can be traced by the rising focus on human actions creating miasma in the doctors’ reports. This ties into how the handling of urban sanitation changed in the 1850s in several Swedish cities, mostly as a response to cholera epidemics. For instance, the emptying of Stockholm’s outhouses had been done by so-called ‘pudrettkäringar’ [women who sold the content as manure]. Many complaints were launched against those handling these tasks, as they were often said to be drunk and spill excrement on the street. In the late 1850s, the city municipality took over the responsibility for emptying latrines, and in the 1870s, sewage pipes were built beneath the streets.^87^

Other historians, such as Tom Koch, have also described this shift, which seems to happen later among Swedish provincial doctors than among medical communities in other countries.^88^ However, this variation in timing does not mean that Sweden is exceptional. Rather, the similarities between national contexts are striking. For instance, the environmental historian Peter Baldwin investigates American discussions on harmful air in the mid-1800s and shows that in the 1870s sewage gas was described as ‘man-made miasma’, citing a housewife’s helper who stated that ‘the matter thrown out of the body, through the lungs and skin, is as truly excrement and in a state of decay as that ejected from the bowels, and as poisonous to the animal system’.^89^ Thus, air which had been contaminated through exhalations and discharges from healthy people – everyday dirt – was described as directly pathogenic. Such air was said to form in theatres, schools, and other places where many people gathered.^90^ Here, the word miasma described matter which during the early nineteenth century would have been regarded only as everyday dirt. Calling dirt caused by everyday living ‘miasmatic’ connected it to risk and turned a practice like overcrowding – previously seen as harmless – into a health hazard. The concept of miasma was heavily charged due to having been used to explain epidemics and death for centuries, and thus, everyday dirt gained a new kind of menace.

Miasmatic matter was rarely described as a medical problem after the mid-1800s. By the end of the nineteenth century, ‘cleanliness’ had increasingly come to mean the act of removing everyday dirt from homes and bodies. ‘Foul smells’, which had previously indicated miasmatic, poisonous vapours, were no longer described as direct health hazards, but were instead only said to be disruptive and unpleasant. Stench had lost its menace. At the same time, however, it became more common to discuss ‘stench’ (‘stank’ in Swedish). In provincial doctors’ reports from the early nineteenth century, the term ‘stench’ was used exclusively to discuss miasmatic fumes. In the late 1800s, the term only described everyday dirt, and its use became very common. In fact, the term was more than a hundred times more frequent in reports from the 1890s than those from the 1830s.^91^

This new kind of medically relevant stench, stemming from everyday dirt, was not said to be the direct cause of disease. Rather, it was used to identify the presence of matter which might become a health hazard in the future. But the way in which it is described reveals that it was perceived as a hybrid between miasma and everyday dirt:

Anyone who knows what a devastating influence the uncleanliness of [poor people’s homes] has on the inhabitants, and who with his nose and lungs can determine the amount of harmful substances in the air, will often realise the need to prescribe cleaning even in places the inhabitants already find clean. The reason is that one gets used to what one sees and senses every day, even dirt…. Eventually, the filth piles up, no fresh air or ray of light can reach it in order to destroy it, water decomposes the putrid substances and the ground absorbs it, thereby becoming completely drenched by pathogenic substances, which are then constantly perspired as harmful vapours.^92^

Here, harmful dirt was identified by its stench, just like miasmatic matter. It also injured people’s health in the same way as miasma did: by means of harmful vapours. What was new is that this kind of dirt originated from healthy bodies, and was possible to get used to. The older type of miasmatic influence had been impossible to adapt to because the body simultaneously sensed and was injured by its stench.^93^ The only kind of dirt one could get used to during the early 1800s was everyday dirt. Thus, the fact that peasants and poor people repeatedly were described as having gotten used to a kind of dirt which was dangerous and which was identified by its stench, is a further sign that a hybrid between everyday dirt and miasma had appeared.

Another sign of the emergence of this hybrid is that doctors began to report sensory overload from everyday dirt. During the 1880s and 1890s, it became common for the provincial doctors to contrast their own shocked reactions to filth to peasants’ uncaring attitudes: ‘the air in peasants’ homes is so bad that someone who is used to breathing fresh air cannot stand it for more than a few moments’, wrote the doctor in Heby in 1898.^94^ Stench was how miasma was identified. Finding health risks by using one’s nose was a remnant from dealing with miasmatic means of disease transmission. The stench from everyday dirt was, however, considered much less dangerous than miasmatic matter – a warning of potential future disease rather than the disease agent itself. After the breakthrough of bacteriology in the late 1870s, dirt was called unhealthy because it contained bacteria; even though sanitary reformers tried to distinguish stench from disease, it remained a powerful warning of an unhealthy environment.^95^ The category of everyday dirt had expanded to incorporate some, but not all, characteristics of the handling of matter believed to cause miasma.

## Everyday dirt becomes pathogenic

It is well known that in many countries, overcrowding turned into a medical problem during the last decades of the nineteenth century. This change can also be observed in the Swedish provincial doctors’ reports. The word ‘crowd’ (‘folksamling’ in Swedish) is used 258 times in the database: eleven times between 1850 and 1879, and 247 times between 1880 and 1900. The word ‘overcrowded’ (‘trångbodd’ in Swedish), was used five times during the 1850s, five times during the 1860s, and 275 times in the 1880s. But *why* overcrowding became a medical problem has not been discussed in great detail, and I suggest that everyday dirt turning into a health hazard contributed to this anxiety. Before dirt generated by healthy bodies began to be considered harmful, overcrowding would not in principle be a medical problem, as long as contagious and miasmatic matter were absent.

Increasing attention to the characteristics of everyday dirt further indicates that it had been charged with a new menace. During the first two-thirds of the century, the smell, texture, and colour of everyday dirt were rarely mentioned. But from the 1870s onwards, places contaminated by everyday dirt began to be meticulously described, as in Söderåkra in 1878:

Chairs, tables and benches are covered in grime, all furniture is filthy and covered with rags scattered about. If one requests a chair, the housewife shoves a few children out of the way, and then quickly wipes her apron or skirt over the seat to cursorily remove some of the dirt. The floor is so covered with spilled substances which have been trampled into the ground that the foot sticks to the floor when you walk on it.^96^

Here, everyday dirt had become such a central object for medical attention that the doctor felt justified in detailing what it consisted of, how the housewife handled it, and how it felt underneath his foot.^97^ This kind of thorough description of filthy homes cannot be found in provincial doctors’ reports from the early 1800s. The verbosity is new. In the same way, instructions for cleaning sick rooms became much more comprehensive during this time. When provincial doctors had described sick rooms before the 1860s, it was most common, even after infectious diseases, to only mention cleaning in passing, and to prescribe it in vague terms, for instance stating only that rooms should be ‘aired out’ and ‘swept’.^98^ In contrast, during the last decades of the century, physicians demonstrated almost encyclopaedic thoroughness.

## Conclusion

In this paper, practical cleaning methods have been investigated as a source of insight into conceptions of disease transmission. The analysis, outlining how Swedish provincial doctors’ perceptions of cleaning and dirt shifted, shows that over the course of the nineteenth century, the medical properties of everyday dirt – bodily emissions from healthy people – changed. Where it used to be seen as a mainly social, aesthetic nuisance, it turned into a pathogenic substance. I have argued that this happened partly through a transfer of properties previously associated with the handling of matter loaded with miasmatic and contagionistic disease agents. Its ability to transmit lethal disease was adopted from both these categories, and the practice of using foul stench to detect everyday dirt was borrowed from how miasmatic matter was handled. Thus, everyday dirt was charged with a pathogenic menace which it previously lacked. This was a precondition for the intense growth of everyday dirt as a medical problem, which was crucial for the momentum of the hygiene movement from the end of the nineteenth century onward.

Thus, towards the end of the century, the cleaning practices of the provincial doctors had come to address a fusion between contagious and miasmatic matter with everyday dirt. This newly threatening everyday dirt became absolutely central to the preventive work of the provincial doctors.

Conceptions of cleanliness are ever-changing. What matter is conceived to be dirt, how dirt affects people’s health, and how a state of cleanliness is achieved, are historically contingent beliefs. Our contemporary notions of what cleanliness entails arose at a specific point in time. In the case of Swedish provincial doctors, that moment was somewhere between 1860 and 1880.

Connecting the newfound menace of everyday dirt towards the end of the nineteenth century with older methods of handling contagious and miasmatic matter in medical practice can hopefully also illuminate nineteenth-century practices beyond the Swedish context. For instance, in *The Gospel of Germs*, Nancy Tomes presents the following two facts as incompatible and puzzling: Although people in the nineteenth century knew, long before germ theory, that those suffering from certain diseases ‘gave off some sort of intangible substance capable of making others sick’, they still ‘shared beds, at home with relatives or in hotels with strangers…. They exchanged combs, hairbrushes, and even toothbrushes, and fed babies from their mouths.… They coughed, sneezed, and spit with blithe disregard for the health consequences to those around them’.^99^ As shown in this article, what Tomes describes should not be seen as surprising. Nonchalance towards proximity to everyday dirt from other people’s bodies, and the acknowledgment that contagious disease was spread by bodily exhalations, were not contradictory at the time, since these responses concerned two separate substances: everyday dirt and contagious matter. Avoiding sick people’s exhalations while disregarding everyday dirt from healthy persons was thus *consistent* behaviour for most of the nineteenth century. It was not until contagious matter and everyday dirt became combined that the thorough medicalisation of cleanliness which Tomes and others have described so well could even happen.

## Footnotes

1 Virginia Smith, investigating cleanliness practices since antiquity, forefronts the late nineteenth century as the period when cleanliness had the greatest societal impact. Virginia Smith , Clean: A History of Personal Hygiene and Purity (Oxford: Oxford University Press, 2007), 266–306 . The same focus can be found in Georges Vigarello , Concepts of Cleanliness: Changing Attitudes in France since the Middle Ages (Cambridge: Cambridge University Press, 1988).

2 Smith*, op. cit.* (note 1); Charles Rosenberg , ‘The Medical Profession, Medical Practice and The History of Medicine’, Edwin Clarke (ed), Modern Methods in the History of Medicine (London: Athlone Press, 1971).

3
*Medicinhistorisk databas* [*Database of Medical History*], Linköping University Electronic Press, https://doi.org/10.3384/db.medhist. For this article, I have read the reports broadly and searched for words related to cleanliness to select approximately a hundred reports where cleanliness practices were discussed in detail, evenly spread over eighty years. The search words were ‘rengöring’ (cleaning), ‘städning’ (cleaning), ‘uppsnyggning’ (tidying up), ‘såpa’ (soap), ‘städa’(cleaning), ‘skrubba’(scrub), ‘vädra’ (air out), ‘röka’ (fumigate),’disinficera’ (disinfect), ‘diska’ (do dishes). All translations from Swedish are my own.

4 By ‘cleaning’ I mean the Swedish words ‘rengöring’, ‘städning’, ‘uppsnyggning’.

5

Christopher Hamlin , Public Health and Social Justice in the Age of Chadwick: Britain, 1800–1854 (Cambridge: Cambridge University Press, 1998); Christopher Hamlin , Cholera: The Biography (Oxford: Oxford University Press, 2009); Christopher Hamlin , ‘Providence and Putrefaction: Victorian Sanitarians and the Natural Theology of Health and Disease’, Victorian Studies, 28 (1985), 381–411 11621267 ; Melanie A. Kiechle , Smell Detectives: An Olfactory History of Nineteenth-Century Urban America (Seattle: University of Washington Press, 2017); Nancy Tomes , The Gospel of Germs: Men, Women and the Microbe in American Life (Cambridge: Harvard University Press, 1999)11623768.

6 For instance, an 1828 English parliamentary investigation into water quality did not connect filthy water to affect the population’s health. Anne Hardy , ‘Water and the Search for Public Health in London in the Eighteenth and Nineteenth centuries’, Medical History, 28 (1984), 250–82, 2506390024 10.1017/s0025727300035936PMC1139446. See also Christopher Hamlin , A Science of Impurity: Water Analysis in Nineteenth Century Britain (Berkeley: University of California Press, 1990). Although some would claim that interest in cleanliness began earlier, for instance, Roy Porter , ‘Cleaning up the Great Wen: Public Health in Eighteenth-Century London’, Medical History, 35 (1991), 61–75 10.1017/s0025727300071118PMC255747411612653.

7

Michael Worboys , ‘Contagion’, Mark Jackson (ed.), The Routledge History of Disease (New York: Routledge, 2018), 76 .

8 The London sanitarian Edwin Chadwick’s 1842 *Report on the Sanitary Condition of the Labouring Classes* was of central importance to this process. Smith, *op. cit.* (note 1), 279; Hamlin, *op. cit.* (note 5).

9

David Arnold , Toxic Histories: Poison and Pollution in Modern India (Cambridge: Cambridge University Press, 2016), 176 .

10

Anne Hardy , Salmonella Infections, Networks of Knowledge, and Public Health in Britain, 1880–1975 (Oxford: Oxford University Press, 2015).

11 Arnold, *op. cit.* (note 9). See also David S. Barnes , The Great Stink of Paris and the Nineteenth Struggle against Filth and Germs (Baltimore: Johns Hopkins University Press, 2006); Michael Worboys , Spreading Germs: Disease Theories and Medical Practice in Britain, 1850–1900 (Cambridge: Cambridge University Press, 2000), 109 , and chapter 4, ‘“Something Definite to Guide You in Your Sanitary Precautions”: Sanitary Science, Poisons and *Contagium Viva*, 1866–1880’, 108–49.

12 Barnes, *op. cit.* (note 11). The same point is made in Christoph Gradmann , ‘Medical Bacteriology: Microbes and Disease, 1870–2000’, Mark Jackson (ed.), The Routledge History of Disease (New York: Routledge, 2018), 381 .

13

Jacob Steere-Williams , The Filth Disease: Typhoid Fever and the Practices of Epidemiology in Victorian England (Rochester: University of Rochester Press, 2020).

14 Hamlin, *op. cit.* (note 5), 3.

15

Yvonne Hirdman , Magfrågan: mat som mål och medel: Stockholm 1870–1920 [*The Stomach Issue: Food as Goal and Means: Stockholm 1870*–1920] (Stockholm: Rabén & Sjögren, 1983).

16

William Bulloch , The History of Bacteriology (Oxford: Oxford University Press, 1960), 129–68. See especially Davaine’s experiments on septicaemia of rabbits, 141–4.

17 Barnes, *op. cit.* (note 11).

18 Tomes, *op. cit.*( note 5).

19

Andrew Wear , ‘The History of Personal Hygiene’, W. F. Bynum and Roy Porter (eds), Companion Encyclopaedia of the History of Medicine (London: Routledge, 1993); Mieneke Te Hennepe , ‘“To Preserve the Skin in Health”: Drainage, Bodily Control and the Visual Definition of Healthy Skin 1835–1900’, Medical History, 58 (2014), 397–442 25045181 10.1017/mdh.2014.30PMC4103382.

20 In 1800, the population was 2.3 million; in 1900, 5.2 million, as stated by Statistiska Centralbyrån [Statistics Sweden], ‘Sveriges folkmängd från 1749 och fram till idag’ [‘The Population of Sweden From 1749 Until Today’], published October 27, 2017, https://www.scb.se/hitta-statistik/artiklar/2017/sveriges-folkmangd-fran-1749-och-fram-till-idag. Accessed February 28, 2022; Karin Johannisson, ‘The People’s Health: Public Health Policies in Sweden’, *Clio Medica*, 26 (1994), 165–82.

21
*Ibid.*

22 Ylva Sjöstrand, ‘Modernitet och traditionalism i svenska provinsialläkarberättelser 1851–1874’ [‘Modernity and Traditionalism in Swedish Provincial Doctors’ Reports, 1851–1874’], *Svensk Medicinhistorisk Tidskrift* [*Swedish Journal for the History of Medicine*], 7 (2003), 139–161; Anna Prestjan , ‘State’s Medical Experts in Local Practice: Provincial Doctors View of Themselves as Public Health Promoters: An Example from the Swedish Countryside, 1880–1920’ Hygiea Internationalis, 9 (2010), 223–46; Peter Nilsson, ‘Folkhälsan i Östskåne under mitten av 1800-talet speglad via en provinsialläkares arbete’ [‘Public Health in Eastern Scania During the Mid-Nineteenth Century Seen Through the Work of a Provincial Doctor’], *Svensk Medicinhistorisk Tidskrift*, 6 (2002), 121–7; Sören Edvinsson , ‘A Country Doctor: Health Care in a mid-Nineteenth–Century Swedish Remote Area’, J. T. H. Connor and Stephan Curtis (eds), Medicine in the Remote and Rural North, 1800–2000 (London: Routledge, 2011).

23 The urbanisation levels in Sweden remained modest during the nineteenth century, only reaching 15.1 per cent in 1880. Johannisson, *op. cit.* (note 20).

24 This is a marked difference from how increased focus on cleanliness in other countries has been described as a reaction to increased urbanisation. This can probably be accounted for by the lack of large cities or extensive industrialised areas in Sweden during this time and shows a remarkable homogeneity, which might be connected to the fact that all provincial doctors were educated together.

25 Between 1814 and 1905, Sweden was in a union with Norway, but the systems of provincial doctors were run separately, as was the Finnish system of provincial doctors until 1809 (when Finland was lost to Russia). In Norway, the system of *distriktslege* was instigated in 1816, see Aina Schiøtz, *Distriktslegen – institusjonen som forsvant: Det offentlige legevesen 1900–1984* [*The District Physician – the Institution which Disappeared: The Medical Profession 1900–1984*] (Oslo: Pax förlag, 2000); Joseph Baroma and Steven Cherry (eds), *Health and Medicine in Rural Europe (1850–1945*) (Valencia: Seminari D’estudis Sobre La Ciència, 2005), 106, 43, 74; Otto E.A. Hjelt, *Svenska och finska medicinalverkets historia 1663–1812: Del II* [*The History of the Swedish and Finnish Medical Board of Health 1663–1812: Part II*] (Helsingfors: Helsingfors Centraltryckeri, 1891), 60–73.

26 A.J. Bruzelius, *Sveriges Läkare-historia Ifrån Konung Gustaf den I:s till närvarande tid* [*The History of Swedish Doctors Since the Reign of King Gustaf I to the Current Time*] (Stockholm: Norstedts, 1895), 601–721.

27 The word ‘God’ (‘Gud’ in Swedish) is mentioned 64 times in the digitised reports, but about half of the words come from the provincial doctor quoting someone else, often to illustrate the fatalism of peasants.

28 Gunnar Broberg, ‘Liten svensk medicinhistoria’ [‘A Short History of Swedish Medicine’], Gunnar Broberg (ed.), *Til at stwdera läkedom: Tio studier i svensk medicinhistoria* [*To Study Medicine: Ten Studies in The History of Medicine in Sweden*] (Stockholm: Sekel, 2008), 9–50.

29
*Ibid.*; Carl Dahlborg, *Svenska provinsialläkarföreningens historia 1881–1905* [*The History of the Swedish Association of Provincial Doctors 1881–1905*] (Stockholm: Svenska tryckeriaktiebolaget, 1930), 20–30.

30
*Ibid.*

31 Hilding Bergstrand, ‘Läkarkåren och provinsialläkarväsendet’ [‘The Medical Profession and the Profession of Provincial Doctors’], *Svenska läkaresällskapet 150 år: Dess tillkomst och utveckling: En återblick* [*150 Years of the Swedish Association of Physicians: Its Emergence and Development: An Overview*] (Lund: Håkan Olsson, 1958), 109.

32 Their fees were fixed, although they were allowed to supplement their income by working as private doctors to local gentry and aristocracy, factory doctors, railway doctors, and so forth. Their state income was meant to supply about half their total annual income. Folder named ‘Afskrifter’ [‘Copies’], *Svenska provinsialläkarföreningens arkiv* [*The Archive of the Swedish Provincial Doctors’ Association*].

33 Bergstrand, *op. cit.* (note 31), 121–9; Bengt Dahlin and Jan Kuuse, *Från provinsialläkare till primärvård: En historisk exposé med exempel från Mittenälvsborg i Västra Götalandsregionen* [*From Provincial Doctor to Primary Care: An Historical Overview with Examples from Mittenälvsborg in the West Gothia Region*] (Lerum: Bengt Dahlin, 2010). *Collegium Medicum* later became *Sundhetcollegium*, in 1877 *Medicinalstyrelsen*, and in the twentieth century was incorporated into what today is *Socialstyrelsen.*

34 Johannisson, *op. cit.* (note 20).

35 Dahlin and Kuuse, *op. cit.* (note 33), 54.

36

Evelyn Ackerman , *Health Care in the Parisian Countryside*, *1800–1914* (New Brunswick: Rutgers University Press, 1990), 8.

37

Pamela K. Gilbert , ‘Popular Beliefs and the Body: “A Nation of Good Animals”’, Michael Sappol and Stephen P. Rice (eds), A Cultural History of the Human Body in the Age of Empire (London: Bloomsbury, 2014), 126 .

38 From Scania in the south to Lapland in the north, Sweden comprises oceanic, humid continental, and subarctic climate zones.

39 The French historian Alain Corbin calls this category of dirt ‘social emanations’, but I prefer the term ‘everyday dirt’ to emphasise its constant accumulation. See Alain Corbin , The Foul and the Fragrant: Odour and the French Social Imagination (Cambridge: Harvard University Press, 1986), 35 .

40 Corbin, *op. cit.* (note 39)*;* Barnes, *op. cit.* (note 11). Concerning Sweden, see Ulrika Graninger, *Från osynligt till synligt: Bakteriologins etablering i sekelskiftets svenska medicin* [*From Invisible to Visible: The Establishment of Bacteriology in Swedish Medicine around the Turn of the Century*] (Stockholm: Carlssons,1997).

41 This overview of the first mention of specific words is not conclusive, since I have not investigated any reports from the eighteenth century. Nor have I compared the statistical occurrence of specific words in the database to the total mass of words. This would be very difficult due to the construction of the database. But this finding well corresponds to other studies of the same period, and to state that there was a lack of medical interest in dirt in the early nineteenth century is hardly controversial.

42 A gap exists in the database between 1870–1877. Therefore, for this quantitative comparison, the reports in the database have been supplemented with forty-sevel reports from 1870–1877, read on microfilm and on photos published on the website of *Riksarkivet.*

43 See for instance the provincial doctor’s report written in 1859 in Uddevalla by Åhlström. Through these three search terms (year, district, author), each referenced report can easily be located in the *Medicinhistorisk databas, op. cit.* (note 3).A gap exists in the database between 1870–1877. Therefore, for this quantitative comparison, the reports in the database have been supplemented with forty-seven reports from 1870–77, in microfilm and photos published on the website of *Riksarkivet.* Including all data in which words occurred when would take up too much room, so I include only one example, indicative of the other described changes. The word dirt (‘smuts’) is used 93 times in the database. The frequency or hits per year is as follows, years not mentioned have 0 hits: 1835: 2, 1841: 1, 1843: 3, 1851: 3, 1852: 1, 1853: 1, 1854: 2, 1857: 1, 1858: 2, 1862: 1, 1864: 2, 1867: 1, 1868: 1, 1878: 3, 1879: 2, 1880: 1, 1881: 4, 1882: 1, 1883: 1, 1884: 3, 1885: 2, 1886: 2, 1887: 3, 1888: 4, 1889: 5, 1890: 6, 1891: 4, 1892: 5, 1893: 6, 1894: 6, 1895: 3, 1896: 3, 1897: 4, 1898: 2, 1899: 1.

44 See for instance 1837 Sigtuna Wistrand; 1835 Vimmerby Hanström.

45 1851 Jönköping Sköldberg.

46 1831 Enköping Rudolphi.

47 1859 Norrtälje Palmgren.

48 1858 Söderåkra Dahlberg. The responsibility for everyday dirt was heavily gendered, as Nancy Tomes and others have pointed out.

49 Annelie Drakman, *När kroppen slöt sig och blev fast. Varför åderlåtning, miasmateori och klimatmedicin övergavs vid 1800-talets mitt* [*When the Open Body Closed. Why Bloodletting, the Miasma Theory and Climate Medicine were Abandoned in the Mid-Nineteenth Century*] (Uppsala, Acta Universitatis Upsaliensis, 2018).

50
*Ibid.*

51 1850 Wrigstad Backström.

52 For similar ideas about the protective value of dirt among French peasants, see Jean-Pierre Goubert , The Conquest of Water: The Advent of Health in the Industrial Age (Princeton, Princeton University Press, 1989).

53 1858 Vimmerby stad Kamph.

54 Corbin, *op. cit.* (note 39), 71.

55 The absence of dirt as a medical concern might raise a question about the relevance of these particular sources. Why would provincial doctors write about dirty bodies in annual reports, meant for medical bureaucrats? However, the same kind of reports from later in the century were filled with descriptions of dirt and of cleanliness practices.

56

Erwin H. Ackerknecht , ‘Anti-Contagionism Between 1821 and 1867’, Bulletin of the History of Medicine 22 (1948), 562–9318106184 .

57

Michael Brown , ‘From Foetid Air to Filth: The Cultural Transformation of British Epidemiological Thought, ca. 1780–1848’, Bulletin of the History of Medicine, 82, 3 (2008), 515–4418791295 10.1353/bhm.0.0070PMC2646604; Smith, *op. cit.* (note 1), 265–9.

58 Worboys, *op. cit.* (note 11).

59 1843 Vimmerby Hanström.

60 In the database, the word ‘disinfection’ can be found for the first time in 1868, 29 times between 1870 and 1879, 220 times between 1880 and 1889, and 939 times between 1890 and 1900.

61 As shown by individual provincial doctors’ private archives.

62 See for instance P.G. Cederskjöld, ‘Till Herrar Provincial-Läkare’ [‘To the Honoured Provincial Doctors’], *Hygiea*, 7, 12 (1845), 379.

63 In an earlier study – *op. cit.* (note 49) – I traced the subscription practices of provincial doctors through the use of surveys gathered by *The Swedish Association for Provincial Doctors* in the 1880s. Lists of subscribers were also occasionally published in medical journals, for instance in *Eira*, 1 (1877). I have read copies of *Eira* from between its founding in 1877 and 1900 and of *Hygiea* from the 1820s onwards while doing other lines of research, and thus know they both follow international developments closely.

64 Broberg, *op. cit.* (note 28), 31.

65 1897 Dorotea Stenberg.

66 See, for instance, 1892 Stockholm Linroth.

67 1900 Nyfors von Post.

68 1881 Naum Grenander.

69 Barnes, *op. cit.* (note 11).

70 1857 Naum Rosling.

71 Ackerknecht, *op. cit.* (note 56).

72 1857 Uppsala Schultz.

73 1859 Kristinehamn Berg.

74 1820 Eskilstuna Alfort.

75 1840 Vimmerby Svensson.

76 1852 Eksjö Bråkenhielm. My italics.

77 Corbin, *op. cit.* (note 39).

78 1857 Uppsala Schultz.

79
*Särskilde komiterades utlåtande angående ordnande af gaturenhållningen m.m. inom hufvudstaden; afgifvet till Öfver-Ståthållare-Embetet den 9 Maj 1862.* [*Statement by the Special Committee Concerning the Arrangement of the Cleaning of the Streets in the Capital: Presented to the Royal Court on May 9, 1862*]. (Stockholm, 1862).

80 1858 Kisa Björck.

81 1853 Enköping Stiegler.

82 1857 Uppsala Schultz.

83 This seems to have happened later in Sweden than in other European countries. For a comparison, see Tom Koch , ‘Mapping the Miasma: Air, Health, and Place in Early Medical Mapping’; Cartographic Perspectives, 52 (2005), 4–27 .

84 1878 Motala Östra Malmberg.

85 1857 Uppsala Schultz.

86 1862 Stockholm stad 1/3 Carlson.

87 Daniel Larsson, *Kolera: Samhället, idéerna och katastrofen 1834* [*Cholera: the Community, the Ideas, the Catastrophe 1834*] (Stockholm: Carlssons, 2015), 188.

88 Koch, *op. cit.*(note 82); Hamlin, *op. cit.*(note 5).

89

Peter C. Baldwin , ‘How Night Air Became Good Air, 1776–1930’, Environmental History, 8 (2003), 412–29, 415.

90
*Ibid.*, 416.

91 The word ‘stench’ occurs once in digitised provincial doctors’ reports from the 1830s, 6 times from the 1840s, eighteen times from the 1850s, four times from the 1860s, two times from the 1870s, fifty-four times from the 1880s, and 195 times from the 1890s.

92 1862 Stockholm stad 1/3 Carlson.

93 Corbin, *op. cit.* (note 39), 89. See, for instance, 1852 Ystads stad Ekman.

94 1898 Heby Lannerstierna; see also 1897 Dorotea Stenberg; 1898 Heby Lannerstierna; 1878 Sigtuna Drake; 1899 Uppsala distrikt von Stapelmohr.

95 Barnes, *op. cit.* (note 11), 3.

96 1878 Söderåkra Dahlberg.

97 It also went from being only a predisposing to both a predisposing and exciting cause for disease. See Christopher Hamlin , ‘Predisposing Causes and Public Health in Early Nineteenth-Century Medical Thought’, Social History of Medicine, 5 (1992), 43–70, 6811612776 10.1093/shm/5.1.43.

98 1838 Helsingborg Schagerström. In Swedish ‘vädrade’ and ‘sopade’.

99 Tomes, *op. cit.*( note 5), 3.

